# Diversity and temporal dynamics of breast milk microbiome and its influencing factors in Chinese women during the first 6 months postpartum

**DOI:** 10.3389/fmicb.2022.1016759

**Published:** 2022-11-11

**Authors:** Bin Liu, Junying Zhao, Yanpin Liu, Weicang Qiao, Tiemin Jiang, Lijun Chen

**Affiliations:** ^1^National Engineering Research Center of Dairy Health for Maternal and Child, Beijing Sanyuan Foods Co. Ltd., Beijing, China; ^2^Beijing Engineering Research Center of Dairy, Beijing Technical Innovation Center of Human Milk Research, Beijing Sanyuan Foods Co. Ltd., Beijing, China; ^3^South Asia Branch of National Engineering Center of Dairy Health for Maternal and Child, Guilin University of Technology, Guilin, China

**Keywords:** breast milk, influencing factor, microbiome, maternal, postpartum, diet

## Abstract

Human breast milk (HBM) plays an important role in providing nutrients, beneficial microorganisms and bioactive components for infants, helping maturation of their immune system and gastrointestinal development. Here, we present a study aiming to investigate the diversity and temporal dynamics of the milk microbiome across the first 6 month postpartum in Chinese healthy breastfeeding women, and to investigate to what extent other variables (e.g., sampling location, infant sex, and mode of delivery) might also be related to variations in the human milk microbiome, and the association with maternal diet and nutrients. Fifty-three healthy pregnant women from four cities were recruited from a China Maternal and Infant Health Cohort Study and breast milk samples were collected and analyzed using 16S rRNA metagenomic sequencing. We illustrated the diversity and temporal dynamics during lactation (Adonis *p*-value = 3*e*–04). Firmicutes and Proteobacteria were the most abundant phyla, and *Streptococcus*, *Staphylococcus*, *Serratia*, and *Corynebacterium* were the core genera. Partitioning around medoids clustering identified two major internal clusters of breast milk microbiota. Cluster 1 was dominated by Acinetobacter and Pseudomonas, while Cluster 2 was dominated by *Streptococcus* and *Staphylococcus*. Among other environmental variables, sampling location showed significant influence on breast milk microbiome (Adonis *p*-value = 4*e*–04), while infant sex (Adonis *p*-value = 0.33) and mode of delivery (Adonis *p*-value = 0.19) were less related to variations in the human milk microbiome. Maternal diet such as tuber was significantly correlated with the relative abundance of Neisseria (rho = 0.34, adjusted *p*-value = 0.01) and Cutibacterium (rho = −0.35, adjusted *p*-value = 0.01), and nutrients such as carbohydrates were significantly correlated with the relative abundance of Aquabacterium (rho = −0.39, adjusted *p*-value = 0.0027), and vitamin B12 was significantly correlated with the relative abundance of *Coprococcus* (rho = 0.40, adjusted *p*-value = 0.0018), etc. These results illustrated the dynamic changes of composition and diversity during the lactation phases of the Chinese breast milk microbiome and addressed the importance of geographic location on milk microbiota, and associations with maternal diet consumption, which have potential benefits on the establishment and future health of breastfeeding infants.

## Introduction

Human breast milk (HBM) is recognized as the optimal and exclusive source of nutrients for infants in the first 6 months of life since it contains simple sugars, lipids, proteins, and many other bioactive components including oligosaccharides, enzymes, cytokines, antibodies, and hormones, which nourish infants and protect them from disease while their own immune system matures (Mastromarino et al., 2014; Li et al., 2017). Breastfeeding protects the infant against infectious diseases such as diarrhea or pneumonia in early life and also has many long-term benefits to the prevention of chronic diseases including allergic diseases, obesity, diabetes, and cardiovascular disease in future life (Binns et al., 2016; Gertosio et al., 2016). HBM provides a great many of beneficial microorganisms that are critical to colonize the mucosal system (including intestinal mucosa) of infant and thereafter contribute to the development and functionality of innate and adaptive immune responses and regulate intestinal barrier homeostasis and nutrient absorption (Hooper and Macpherson, 2010; Mastromarino et al., 2014; Ojo-Okunola and Nicol, 2018). Results of several studies have shown the beneficial effects of commensal bacteria in HBM on the newborn infants (Heikkila and Saris, 2003; Pérez-Cano et al., 2010; Pannaraj et al., 2017; Biagi et al., 2018).

The presence of bacteria in HBM as a result of natural selection has made it uniquely appropriate for nourish infants (Goldman, 2012). About 10^3^–10^5^ cfu/ml viable bacteria were identified in HBM from healthy women by using culture-dependent method (Perez et al., 2007), and an infant who consuming an average of 800 ml/day HBM has been reported to intake 10^4^–10^6^ commensal bacteria (Heikkila and Saris, 2003). Culture-dependent methods have long shown the predominance of the lactic acid bacteria including species of *Lactobacillus* and *Bifidobacterium* (Martín et al., 2003), whose productions would improve the health of gastrointestinal tract through limiting the growth of potential pathogenic organisms. More recently, a more complex bacterial community were detected by using culture-independent DNA-based techniques such as next-generation sequencing, including *Bifidobacterium* spp., *Bacteroides* spp., and members of the Clostridia class (Hunt et al., 2011; Fouhy et al., 2012). The most abundant phyla were found to be Firmicutes and Proteobacteria (Cabrera-Rubio et al., 2016; Kumar et al., 2016), and *Streptococcus*, *Staphylococcus*, *Serratia*, and *Corynebacterium*, as the members of dominant “core” bacterial genera which were reported to be less influenced by the environmental factors (Hunt et al., 2011). However, various studies have reported differences in this “core” bacterial genera in different countries by using different subjects, sample collection, and detection methods (Hunt et al., 2011; Urbaniak et al., 2016; Li et al., 2017).

Various studies have confirmed that nutritional component in HBM varied across different stages of lactation period accompanying with the change of physiological, hormonal, and pathological conditions of breastfeeding women, especially for colostrum and transitional milk (Jiang et al., 2016; Xue et al., 2017; Bai et al., 2018). However, it was controversial that the variation of microbiota in HBM was associated with lactation stages (Cabrera-Rubio et al., 2012; Khodayar-Pardo et al., 2014; Sakwinska et al., 2016; Li et al., 2017), possibly due to lack of enough high-quality samples with reasonable sampling time points from cohort study. Additionally, many other factors have been shown to be associated with the differences of bacteria in HBM, including delivery mode (Cabrera-Rubio et al., 2012; Khodayar-Pardo et al., 2014; Cabrera-Rubio et al., 2016), gestational age (Khodayar-Pardo et al., 2014), maternal weight (Cabrera-Rubio et al., 2012; Collado et al., 2012), maternal health [allergy (Grönlund et al., 2007), celiac disease (Olivares et al., 2015), or HIV (González et al., 2013)], geographical location (Kumar et al., 2016; Li et al., 2017), and usage of antibiotic (Soto et al., 2014). Breast milk fatty acids and minerals (Ca and Mg) appeared to influence microbial composition of HBM (Sanjulián et al., 2021). Cabrera-Rubio et al. (2016) found that milk microbiome from mothers with vaginal delivery clearly separated from those with C-section, and milk from obese women showed a different and less diverse microbiota when comparing with milk from normal-weight women. However, the association of pregestational BMI and gestational weight gain (GWG) with bacterial community in HBM has been little investigated.

We hypothesized that the change of physiological or hormonal triggers during lactating period which influenced breast milk components may also support different bacterial genera. This prospective, observational cohort study was designed primarily to evaluate the diversity and temporal dynamics of the milk microbiome across the first 6 month postpartum in Chinese healthy breastfeeding women. Second, we investigated to what extent other variables (e.g., sampling location, maternal age, pregestational BMI, gestational weight gain, maternal diet, infant sex, and delivery mode) might also be related to variations in the human milk microbiome.

## Materials and methods

### Study population

A representative subset of 53 mother was selected from China maternal and infant nutrition health cohort study (MINC), a prospective cohort study focuses on the effect of early life nutrition and diet on the short- and long-term health of infants in China (Jiang et al., 2018). Health pregnant women with the age of 18–40 were enrolled between 2014 and 2017 (*n* = 962) and remained eligible if they delivered a healthy term infant (37–40 weeks gestation) with a birth weight range from 2,500 to 4,000 g appropriate for gestational age (*n* = 648). In the current study, we selected a representative subset of 53 mothers with available milk samples at least three sampling location, ensuring equal representation across the 5 phases of 0–6 months. The study was approved by Ethics Committee of Beijing Ditan Hospital affiliated to Capital Medical University (#2015-027-01), and informed written consent was obtained from all mothers.

### Human breast milk sample collection

A total of 204 human breast milk samples were obtained from 53 healthy lactating women (mean age 29.8 ± 3.7, range 22–38). The sample numbers collected from each city and lactation phase are shown in Table 1. Breast milk sampling was standardized for all subjects, and was performed in a dim light room in hospitals without direct sunlight exposure. An electric pump (Horigen HNR/X-2108ZB, Xinhe Electrical Apparatuses Co., Ltd., Guangzhou, China) was used to collect the milk. Breasts of the subjects were emptied by the mother herself between 6 and 7 a.m. After the mothers had breakfast (7–8 am), the samples were collected at the second feeding in the morning (9–11 am) to avoid circadian influence on the outcomes. A single full breast was emptied by trained investigators into a new feeding bottle. We attempted to collect the milk from the same (right) breast, but milk was also collected from the left side when a steady stream of milk was not possible for some mothers. After gently up–down shaking for ~ 10 times, an aliquot of 10 ml was secured for characterization purposes. The rest of the milk was returned to the mother for feeding to the infant. Each sample was distributed in 5 ml clear polypropylene tubes under the dim light on ice, labeled with subject information, and stored at −80°C. Once producing, the HBM samples were delivered to the laboratory immediately to ensure that all of them would arrive at the laboratory within one or 2 days after collection, and then stored at −80°C until DNA extraction. HBM samples were collected at 0–5 days (colostrum), 12–14 days (transitional milk), 1, 4, and 6 months after delivery (defined as different phases range from S1 to S5). The information of those lactating women was collected including age, height, pregestational weight, prepartum weight, and postpartum weight. Additionally, mode of delivery, pregnancy weeks, sex, and birth weight of infants were recorded when delivery.

**Table 1:** Sample numbers of human breast milk by city and lactation phase.

Phase/city	Beijing	Liuyang	Luoyang	Tangshan	Subtotal
3–5 days (S1)	19	5	10	5	39
13–15 days (S2)	22	6	11	5	44
1 month (S3)	25	8	12	6	51
4 months (S4)	19	8	7	5	39
6 months (S5)	16	4	7	4	31
Subtotal	101	31	47	25	204

### Diets and nutritional components

Maternal 3-day-diet (before collection of breast milk samples) was recorded using a 32-diet-item food frequency questionnaire (FFQ) as described in Zhang et al. (2021). Diet intake, such as tuber, vegetable, fruit, meat, fish, egg, milk, soy, oil, and nutritional components, such as energy (Kcal), protein (g), fat (g), carbohydrates (g), fiber (g), cholesterol (mg), vitamin A, vitamin B1, vitamin B2, vitamin B6, vitamin B12, folate (vitamin B9), niacin (vitamin B3), vitamin C, vitamin D, vitamin E, calcium (Ca), phosphorus (P), potassium (K), sodium (Na), magnesium (Mg), ion (Fe), zinc (Zn), selenium (Se), cuprum (Cu), manganese (Mn), and iodine (I), were analyzed according to Chinese Dietary Reference Intakes (DRIs) using Nutrition Calculator v2.7.3.12.^1^

### High throughput 16S rRNA amplicon sequencing

Genomic DNA was extracted from 5 ml of homogenized milk samples and reagent control samples using the QIAamp Fast DNA Stool Mini Kit (Qiagen, GmbH, Hilden, Germany) following the manufacturer’s protocol. PCR amplification of the V3–V4 region of 16S rRNA genes (primers 341F: CCTACGGGNGGCWGCAG and 805R: GACTACHVGGGTATCTAATCC) was performed with 10 ng DNA as a template, using 15 μl of Phusion High-Fidelity PCR Master Mix (New England Biolabs), 0.2 μM of forward and reverse primers in a 30 μl total reaction volume. The PCR program included 3 min at 95°C, 25 cycles of 30 s at 95°C and 30 s at 55°C, and then 30 s at 72°C. Sequencing libraries were generated using NEB Next Ultra DNA Library Prep Kit for Illumina (NEB, United States) following manufacturer’s recommendations. Reagent control samples were failed to pass library quality check due to insufficient DNA concentration. Amplicons were sequenced using 2 × 250 bp paired-end by Illumina HiSeq 2500 according to the manufacturer’s instructions.

Breast milk microbiome data analysis was performed using USEARCH (version 11.0.667; Edgar, 2010) and VSEARCH (version 2.22.1; Rognes et al., 2016), the demultiplexed raw paired-end reads were quality filtered, clustered, and removed chimeras. The operational taxonomic units (OTU) was generated with 97% similarity cutoff, and then the taxonomy ranks were assigned to the representative sequences using the RDP 16S rRNA training set (Version 18, latest release on 2020-August-14; Wang et al., 2007), which includes the reclassification of the genus Lactobacillus into 25 genera (Zheng et al., 2020) and other taxonomy updates.

### Statistical analysis

#### Species diversity

α-diversity indexes (Chao1 and Shannon) were calculated at OTU level using Vegan package in R based on the OTU level abundance profile. Differences of α-diversity indexes were tested using Wilcoxon Rank sum test. Phylogenetic measures of β-diversity based on the genus level abundance profile were also calculated by using Vegan package and Principal Coordinate Analysis (PCoA) plot analysis was displayed by WGCNA package, stat packages, and ggplot2 package in R software. We used Adonis of Bray–Curtis distances to compare the microbiota community structure across phases and groups. Differential abundance of genera and phyla was tested by Wilcoxon rank sum test, and *p* values were corrected for multiple testing with the Benjamin & Hochberg method. Linear discriminant analysis (LDA) of effect size (LEfSe) was applied to determine the most discriminant taxa among phases and cities (LDA score ≥ 4.0 and p value < 0.05) using microeco package (Liu et al., 2021) in R. R software (Version 4.2.1) which was used for plotting and statistical analysis throughout.

## Results

### Description of cohort

A total of 204 human milk samples from 3 to 5 days (*n* = 39), 13–15 days (*n* = 44), 1 month (*n* = 51), 4 months (*n* = 39), and 6 months (*n* = 31) after delivery were obtained from 53 mothers selected in this cohort study. The average age of subjects was 29.8 ± 3.7 years (range 22–38 years) and the mean pregestational BMI was 21.2 ± 2.9 kg/m^2^ (range 16.7–27.4 kg/m^2^). According to the recommendation by Institute of Medicine, 44 (83.0%), 8 (15.1%), and 1 (1.9%) participants had insufficient, appropriate, and excessive gestational weight gain (GWG), respectively. The mean birth weight of their infants was 3514.5 ± 470.7 (range 2,500–4,700 g) and the mean birth body length was 50.7 ± 1.5 cm (range 46–54 cm). Twenty-eight (52.8%) infants were male and 33 (62.3%) infants were delivered by natural childbirth. The detailed information of the participants’ and their infants’ characteristics are displayed in Table 2.

**Table 2:** Baseline characteristics of participants and infants (*n* = 53).

Characteristics	Mean ± SD/n (%)
Mothers	
Age (years)	29.85 ± 3.66
Height (m)	1.63 ± 0.04
Pregestational weight (kg)	56.62 ± 8.50
Prepartum weight (kg)	71.19 ± 9.78
Postpartum weight (kg)	64.05 ± 9.53
Pregestational BMI (kg/m^2^)	21.45 ± 2.91
Gestational weight gain	
Insufficient	44 (83.00)
Appropriate	8 (15.10)
Excessive	1 (1.90)
Infants	
Gender, *n* (%)	
Male	28 (52.79)
Female	25 (47.21)
Birth weight (g)	3534.04 ± 453.17
Birth body length (cm)	50.69 ± 1.49
Delivery mode, *n* (%)	
Natural delivery	33 (61.50)
Cesarean delivery	20 (38.50)

### Microbiota diversity and composition varied by sampling time

**Figure 1:**
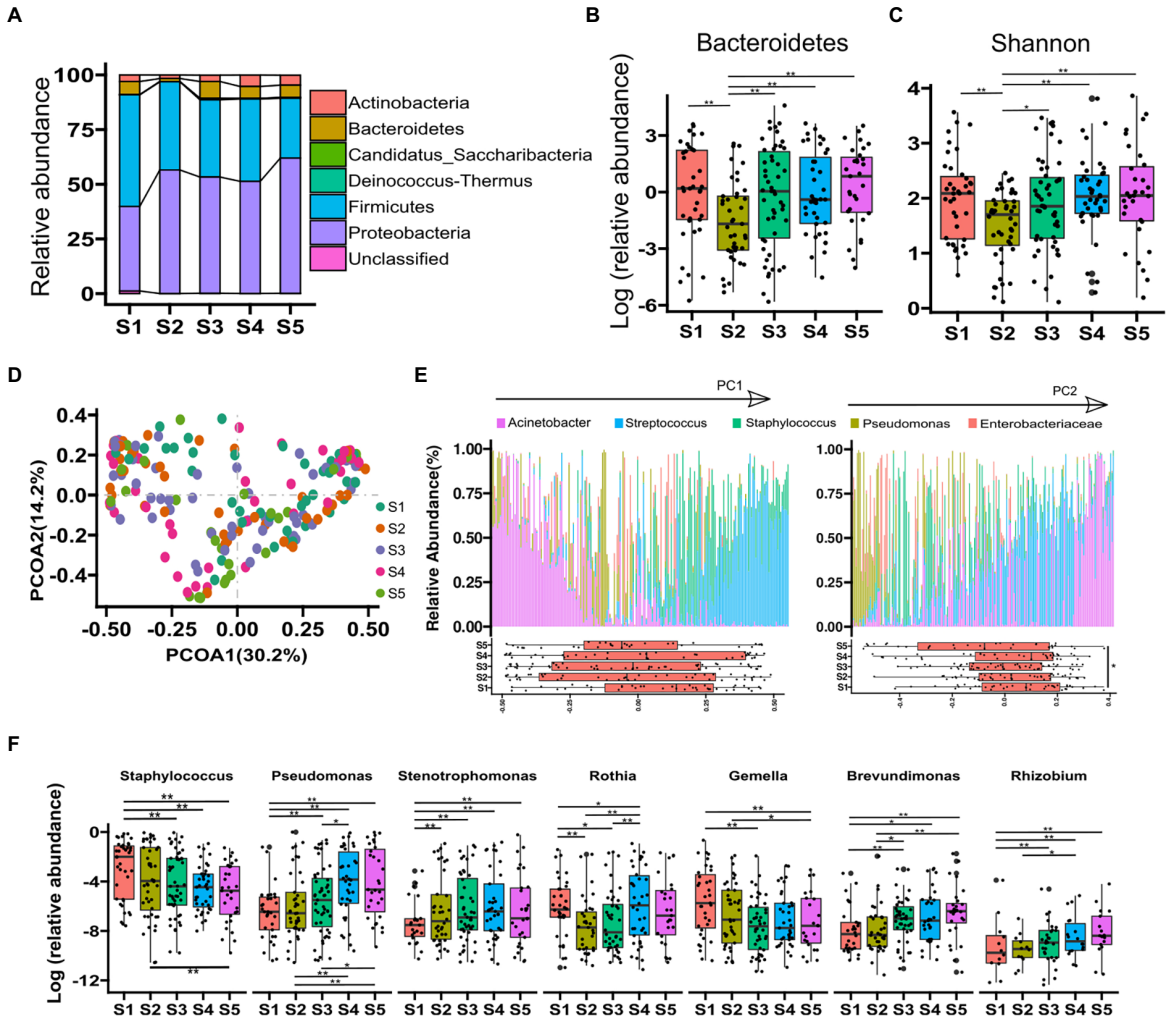
Microbiota diversity and composition varied by lactation phase. **(A)**, the relative abundance of phyla in each phase. **(B)**, statistical analysis of Bacteroidetes from each phase. **(C)**, difference of Shannon index of samples from all phases. **(D)**, principal coordinates analysis (PCoA) using relative abundance data at genus level. **(E)**, five predominant genera in the 203 breast milk samples. The most abundant bacterial genera are indicated by arrows and genus names. Two principal components (PC1 and PC2) were showed. Boxplots showed the variance of microbial abundance at each stage. **(F)**, boxplot of the top 7 species from all phases and MetaStat by permutation test [*p* value was the adjusted result of Benjamini and Hochberg False Discovery Rate (BH FDR)]. ^*^p < 0.05, ^**^p < 0.01.

A total of 13,718,055 high-quality filtered reads were obtained, and the reads were clustered into 1,377 OTUs. The OTUs were then assigned to known taxa (27 phyla, 49 classes, 81 orders, 150 families, and 293 genera). The 4 predominant phyla detected were Proteobacteria (52.3%), Firmicutes (38.4%), Bacteroidetes (5.2%), and Actinobacteria (3.5%), and the component ratio of phyla in each stage is shown in Figure 1A. The abundance of Bacteroidetes was significantly lower in the samples at stage 2 compared with the other phases (all *p*-value < 0.05; Figures 1B). α-diversity (Shannon index) of human milk microbiome at stage 2 was significantly lower than stages 1, 4, and 5 (all *p*-value < 0.05; Figures 1C). PCoA based on Bray–Curtis dissimilarity at genus level was performed to identify the association of microbial composition in different lactation phases (Figure 1D). PC1 and PC2 explained 44.9% of the variance. PC1 was enriched with *Acinetobacter*, *Streptococcus*, and *Staphylococcus*, whereas PC2 was enriched with Pseudomonas. The box plots in Figure 1E showed that samples from different phases were allocated in the PC2-postive region (*p*-value < 0.05 for stage 1 vs. stage 5). β-diversity distances of different phases were compared, and we found that microbial community structure of samples at stage 1 was significantly varied from other phases (Adonis *p*-value < 0.05), and stage 2 was significantly varied from stage 4 and 5 (Adonis *p*-value < 0.01). We also explored the association of the 20 most predominant bacterial genera with lactation phases, and found that the relative abundances of predominant genera, such as *Staphylococcus*, *Pseudomonas*, *Stenotrophomonas*, *Gemella*, *Rothia*, *Rhizobium*, and *Brevundimonas* were significantly varied with lactation phases (all adjusted *p*-value < 0.05, Figure 1F). Among them, there was a significant decrease in abundance of *Staphylococcus* and *Gemella* along with lactating stage, while an increase in *Pseudomonas* and *Stenotrophomonas* (Figure 1F). Other predominant genera such as *Acinetobacter*, *Streptococcus*, *Enterobacteriaceae*, *Enhydrobacter*, *Serratia*, *Chryseobacterium*, *Bacteroides*, *Enterococcus*, *Sphingobacterium*, *Faecalibacterium*, *Prevotella*, and *Corynebacterium* showed no significant change in different lactation phases (adjusted *p*-value > 0.05).

### The clusters identified in human milk and their association with characteristics of lactating women

**Figure 2:**
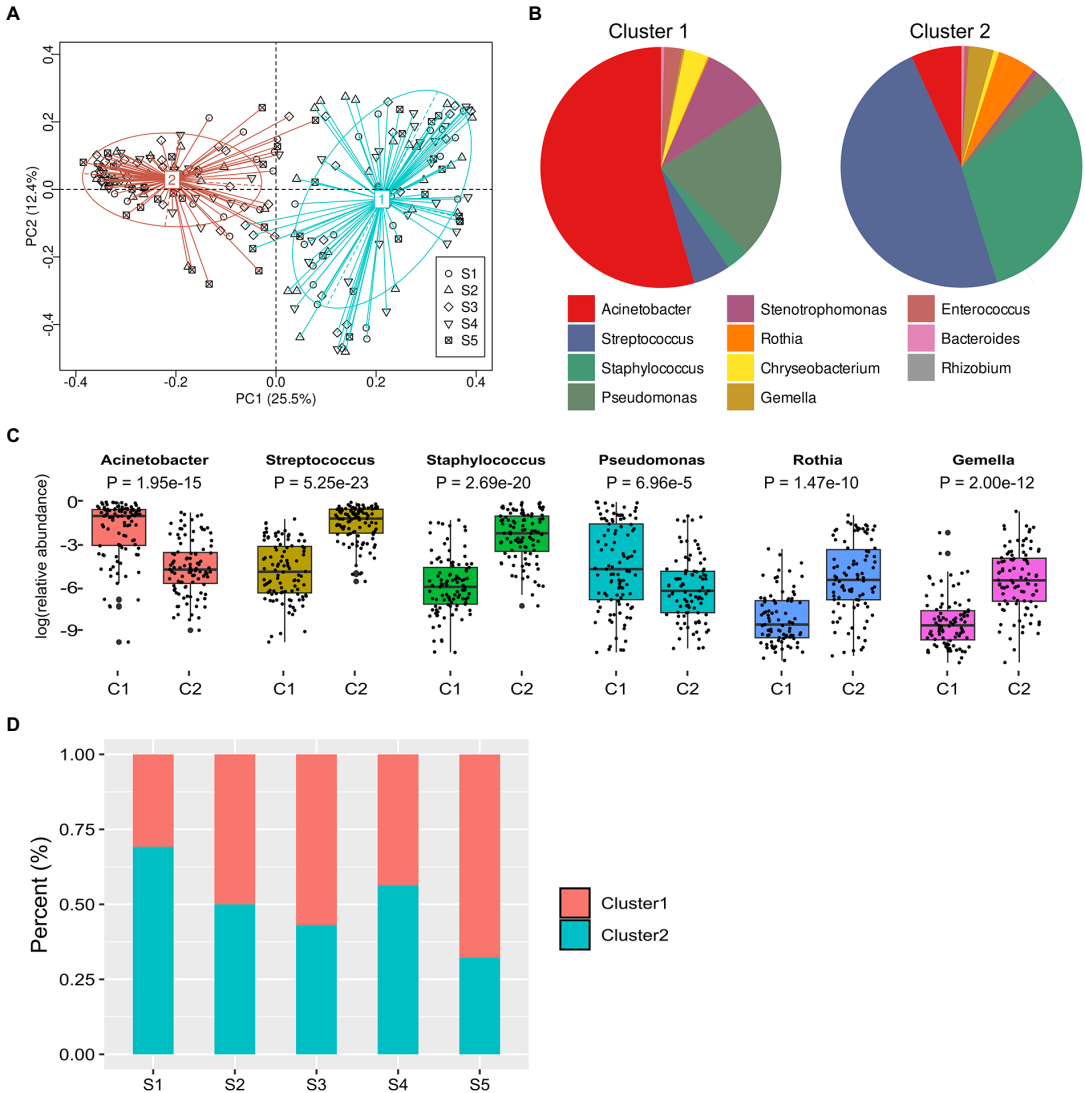
Partitioning around medoids (PAM) clustering and analysis of milk microbiome at different lactation phases. **(A)**, Principal Coordinate Analysis (PCoA) on the relative abundance data of clusters at different lactation phases. **(B)**, distribution of the top 15 predominant genera in two clusters. **(C)**, boxplot of the top 4 species from two clusters and MetaStat by permutation test [*p* value was the adjusted result of Benjamini and Hochberg False Discovery Rate (BH FDR)]. ^*^p < 0.05, ^**^p < 0.01. **(D)**, percentage of two clusters at lactation phases.

We figured out the taxonomic clustering of samples by PCoA and the partitioning around medoids (PAM) clustering at the genus level, and the optimum cluster number was two, which was identified based on the Calinski–Harabasz (CH) index (Figure 2A). Our results showed that Cluster 1 was driven by the genera *Acinetobacter* and *Pseudomonas* (*p* < 0.05), and Cluster 2 was driven by the genera *Streptococcus* and *Staphylococcus* (*p* < 0.05, Figure 2B). Among the 20 predominant genera, the relative abundances of *Acinetobacter* and *Pseudomonas* were significantly higher in Cluster 1 than those in Cluster 2 (all adjusted *p*-value < 0.05), whereas the abundances of *Streptococcus*, *Staphylococcus*, *Rothia*, and *Gemella* in Cluster 2 were significantly higher than those in Cluster 1 (all adjusted *p*-value < 0.01; Figures 2C). We further analyzed the enrichment of clusters in each stage and found that the percentage of Cluster 2 was decreased along with lactation phases, and the percentage of Cluster 2 at stage 1 was significantly higher than it at stage 3 or 5 (both *p*-value < 0.05, Figure 2D).

### Microbiota difference among cities

The 204 human milk samples were obtained from 53 healthy lactating mothers living in four cities of Beijing, Luoyang of Henan province, Liuyang of Hunan province, and Tangshan of Hebei province. No significant differences were found in mothers’ age or BMI, and in baby gender among the regions. For delivery mode, no significant differences were observed among Beijing, Luoyang, and Liuyang, which was significantly different from Tangshan with vaginal delivery only.

**Figure 3:**
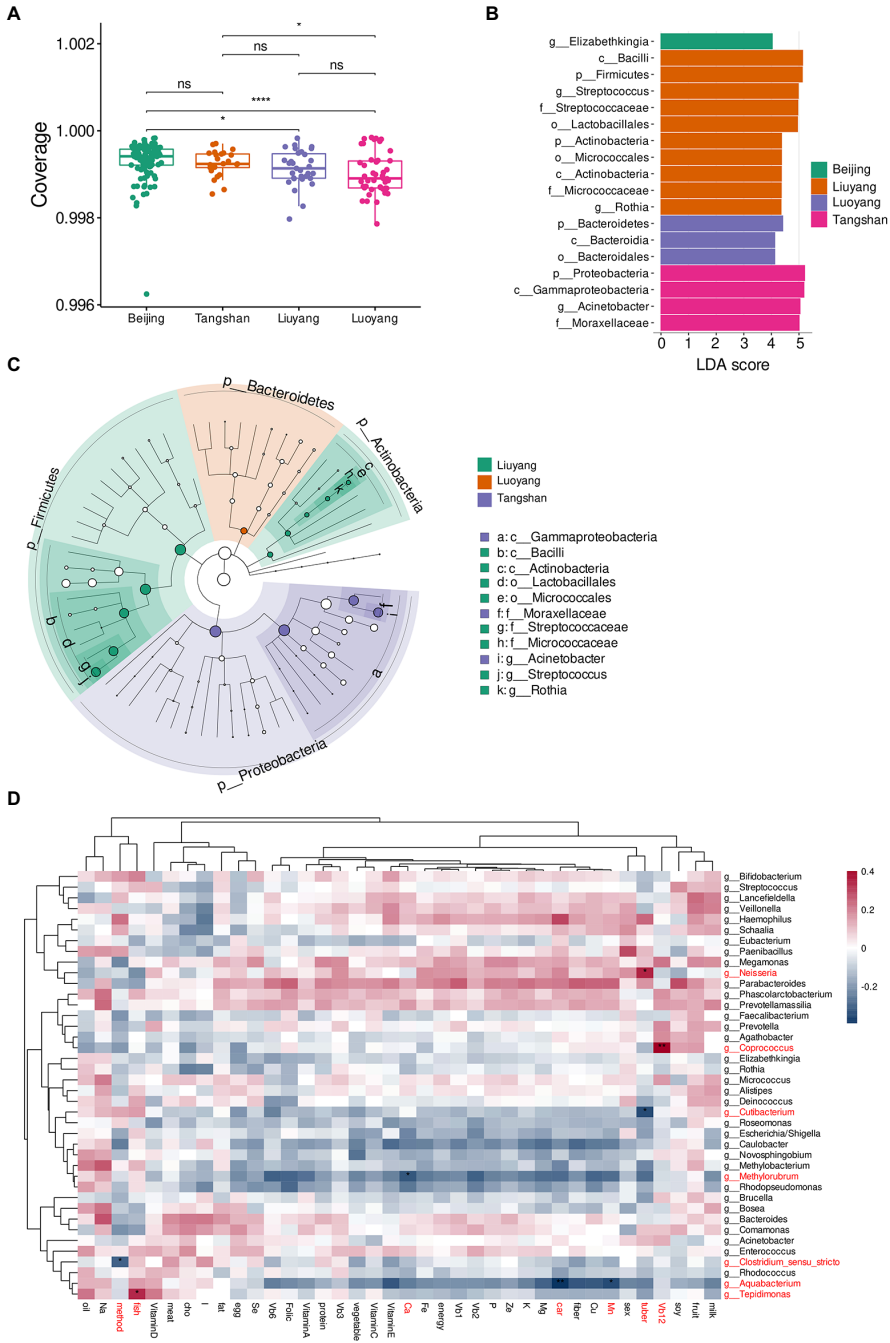
Comparison of milk microbial composition in different cities. **(A)**, α-diversity (observed species) based on the OTUs in Beijing **(B)**, Luoyang (L), Liuyang (H), and Tangshan (T). LEfSe barplot (B) and cladogram **(C)** of LDA value for Taxa with significant difference (LDA Score > 4, *p* < 0.05) between among cities. ^*^p < 0.05; ^**^p < 0.01, ^***^p < 0.001. **(D)**, association heatmap of maternal diet and nutritional components with microbiome of breast milk, diet intake such as tuber, vegetable, fruit, meat, fish, egg, milk, soy, oil, and nutritional components such as energy (Kcal), protein (G), fat (g), carbohydrates (g), fiber (g), cholesterol (mg), vitamin A, vitamin B_1_, vitamin B_2_, vitamin B_6_, vitamin B_12_, folate (vitamin B_9_), nacin (vitamin B_3_), vitamin C, vitamin D, vitamin E, calcium (Ca), phosphorus (P), potassium(K), sodium (Na), magnesium (Mg), ion (Fe), zinc (Zn), selenium (Se), cuprum (Cu), manganese (Mn) and iodine (I). Color of cell stands for Spearman’s correlation coefficients. ^*^p < 0.05, ^**^p < 0.01.

The species diversity analysis of milk microbiota from different cities showed that α-diversity (Coverage index) significantly differed among Beijing, Liuyang and Luoyang, and also differed between Tangshan and Luoyang (Wilcoxon rank-sum test; Figure 3A). We further compared the microbiota community structure based on the Bray–Curtis distances by Adonis analysis, founding no significant difference among the Northern cities of Beijing, Luoyang, and Tangshan, but significantly different from southern city of Liuyang (*p*-value < 0.05). This difference could be associated with the most discriminant taxa determined by LEfSe analysis, such as Phylum of Proteobacteria, Class of Gammaproteobacteria, and Genus of Acinetobacter riched in samples from Tangshan, genus Elizabethkingia riched in those from Beijing, and Phylum of Firmicutes and Actinobacteria, genus of *Streptococcus*, Rothia, and so on riched in Liuyang, and Phylum of Proteobacteria, Class of Bacteroidales riched in Luoyang (Figures 3B,C). Other factors such as gender of infant (Adonis *p*-value = 0.33) and delivery methods (Adonis *p*-value = 0.19) showed limited influence on human milk microbiome.

### Association of maternal diet and milk components with microbiome of breast milk

Spearman correlations of human milk microbes with thirty-two maternal diet components and other factors (infant sex and delivery mode) were performed.. For diet intake, Neisseria and Cutibacterium were significantly correlated with tuber (rho = 0.34, adjusted *p*-value = 0.01; rho = −0.35, adjusted p-value = 0.01, separately); for nutrients, Aquabacterium was significantly correlated with carbohydrates (rho = −0.39, adjusted *p*-value = 0.0027), and Tepidimonas was significantly correlated with fish (rho = 0.34, adjusted *p*-value = 0.029), Coprococcus was significantly correlated with vitamin B12 (rho = 0.40, adjusted *p*-value = 0.0018), Methylorubrum was significantly correlated with Ca (rho = −0.32, adjusted *p*-value = 0.049), and Aquabacterium was significantly correlated with Mn (rho = −0.34, adjusted *p*-value = 0.024). Also, Clostridium *sensu stricto* was significantly correlated with delivery method (rho = −0.30, adjusted *p*-value = 0.039). Other diets and nutrients showed no significant correlation with milk microbial genera (adjusted *p*-value>0.05; Figure 3D).

## Discussion

Human breast milk (HBM) is known as the fundamental origin of nutrition including bacteria for neonate in the first 6 months of life that nourish infants gastrointestinal and immune development and protect them from diseases (Mastromarino et al., 2014; Li et al., 2017) and regulate intestinal barrier homeostasis and nutrient absorption (Hooper and Macpherson, 2010; Mastromarino et al., 2014; Ojo-Okunola and Nicol, 2018). Although various studies have confirmed that nutritional component in HBM varied across different phases of lactation period accompanying with the change of physiological, hormonal, and pathological conditions of breastfeeding women, especially for colostrum and transitional milk (Jiang et al., 2016; Xue et al., 2017; Bai et al., 2018), it was still controversial that the variation of microbiota in HBM was associated with lactation phases and other environmental factors (Cabrera-Rubio et al., 2012; Khodayar-Pardo et al., 2014; Sakwinska et al., 2016; Li et al., 2017).

Using 16S rRNA amplicon sequencing technology to investigate the microbiome of human milk across the first 6 month postpartum in Chinese healthy breastfeeding women in this prospective, observational cohort study, we illustrated the diversity and temporal dynamics of the human milk microbiome, and also found that environmental factors (lactation phase and city) were related with the microbial variations. These results provide new information about human breast milk microbiome and its impact factors, and provide potential aspects of modulate milk microbiome composition.

Predominant bacterial phyla and genera were consistent with published results (Hunt et al., 2011; Cabrera-Rubio et al., 2016; Kumar et al., 2016), as they found that the most abundant phyla were Firmicutes and Proteobacteria (Wan et al., 2020), and core genera (less affected by the environmental factors) such as Streptococcus, Staphylococcus, Serratia, and Corynebacterium. However, various studies reported different “core” genera from different countries with different individuals, sampling methods, and test methods (Hunt et al., 2011; Urbaniak et al., 2016; Li et al., 2017). Predominant genera Staphylococcus showed high relative abundance in early lactation phases, and then decreased, which is consistent with previous studies (Urbaniak et al., 2016; Wan et al., 2020), and this may associated with early colonization of breastfeeding neonatal gut microbiota. Using PAM clustering algorithm to illustrate internal patterns of breast milk microbiota, we identified two major clusters. Cluster 1 was dominated by Acinetobacter and Pseudomonas, while Cluster 2 was dominated by Streptococcus and Staphylococcus. The percentage of Cluster 1 was decreased during lactation phases could confirm the belief that the microbes of HBM may meet the requirements of the infant in each lactation stage. Previous study showed that there were three clusters in milk microbiota of Chinese women, and the major key bacterial families were Streptococcaceae (abundance in Cluster 2, 48.5%), Staphylococcaceae (abundance in Cluster 1, 42.1%), and Pseudomonadaceae (abundance in Cluster 3, 26.5%)(Li et al., 2017). Moossavi et al. (2019) found four main clusters in the milk microbial community, and C1 was dominated by Enterobacteriaceae, Moraxellaceae, and Pseudomonadaceae, while C2 was dominated by Streptococcaceae and Staphylococcaceae, which may partially be influenced by transit from oral to exogenous bacteria. Differences of cluster number in different studies may due to varied parameters of clustering methods, breast milk collection methods and geographic diet and life style variations. Lactation stage has been recognized as a factor influencing breast milk microbes (Cabrera-Rubio et al., 2012), and it has been shown that specific breast milk microbial genera such as Staphylococcus, Streptococcus and other microbes could be transmitted to infants and beneficial for the infant gut (Benito et al., 2015; Fehr et al., 2020). This could reinforce the belief that the microbes of HBM may affect the infant’s early intestinal colonization and meet the requirements for the infant in each lactation period.

As reported previously, environmental variables (e.g., sampling location, infant sex, and delivery mode) were related with variations in the human milk microbiota (Cabrera-Rubio et al., 2012; Sakwinska et al., 2016; Li et al., 2017). Sample geographic location, which influences the long-term maternal dietary choices and other lifestyles, is reported as one of the factor that can influence microbiota of human breast milk. Milk microbiota was highly geographical location specific, as Lactobacillus was high in Northwest and North China, and *Lactobacillus reuteri* was high in Tibet and Gansu provinces (Ding et al., 2019). Other study also showed that lactation phase was the predominant factor in dynamic changes of milk microbiome (Sanjulián et al., 2021), but delivery mode does not influence microbial changes of human milk during lactation (Lyons et al., 2022).

Microbial composition and diversity of milk were related with other maternal factors, such as BMI, parity, delivery mode, and infant sex, and especially, breastfeeding practices were identified as the key determinant of milk microbiota composition (Moossavi et al., 2019). The number of unique OTUs was significantly greater in the breast milk from Cesarean section group than from vaginal delivery group (Li et al., 2017), and microbiome of breast milk from elective cesarean delivery showed a significant compositional change compared with vaginal delivery and nonelective cesarean delivery (Cabrera-Rubio et al., 2012). In this cohort study, adonis analysis showed that lactation stage was the major factor, followed by sampling sites; other factors such as gender of infants and delivery methods showed limited influence on human milk microbiota. Studies have shown that other factors such as maternal diet and nutrients of HBM can also modulate and shape the microbial composition and diversity of breast milk (Mazurier et al., 2017; Cortes-Macías et al., 2021).

Diet provides nutritional components to breast milk, and it is recognized as one of the major factors that could influence breast milk microbiome, as reported by several studies (Ferraris et al., 2020; Redondo-Useros et al., 2020; Sanjulián et al., 2021). In this study, tuber consumption in diet was significantly correlated with Neisseria and Cutibacterium. Neisseria was reported to be related with infectious diseases (Wang and Donovan, 2015); Cutibacterium, which was sourced from the skin, was found a higher relative abundance in Spain, while Aquabacterium, who was significantly correlated with carbohydrates consumption in diet, was reported as a differential genera that was higher in Burundi (Kumar et al., 2016; Drago et al., 2017), and could transfer to breast milk and then to the infant gut (Jost et al., 2014; Zimmermann and Curtis, 2020). According to Yang et al. (2020), carbohydrates, especially dietary fibers, were related with increase of several health beneficial gut microbes, such as *Bifidobacterium*, *Faecalibacterium*, *Lactobacillus*, and *Akkermansia*, etc. *Coprococcus*, who was significantly correlated with diet vitamin B12 consumption, was reported to be vertically transmitted from mother to infant (Asnicar et al., 2017). Several micronutrients, such as vitamins and minerals, were also related with a healthy gut microbiome in appropriate supplements (Yang et al., 2020). Apart from diet in this study, gut microbial genera Clostridium *sensu stricto* was significantly correlated with delivery methods, which previously showed higher abundance in full-term caesarean section group compared to full-term spontaneous vaginally delivered group at first week after born in infant gut (Hill et al., 2017).

Although reagent controls were tested, other reference or mock community were not included in our library preparation and sequencing pipeline, as suggested by previous review (Hornung et al., 2019), so we cannot rule out potential contaminants. Also, shotgun metagenomic sequencing is needed to illustrate the strain level and biological functions of gut microbes, and culturing experiments, especially culture of probiotics in human breast milk, are required to fully reveal the existence and biological characteristics of them.

## Conclusion

In this prospective, observational cohort study, we have used 16S rRNA amplicon sequencing technology to investigate the microbiota of human milk across the first 6 month postpartum in Chinese healthy breastfeeding women. Our results showed the diversity and temporal dynamics of human milk during lactation, and environmental variables such as lactation stage and sampling location are major influencing factors in the human milk microbiome, and the association with maternal diet and nutrients. Further studies are needed to illustrate how breast milk microbiome impact establishment and maturation of infant’s gut microbiome and future health.

## Data availability statement

The data presented in the study are deposited in the Figshare repository, DOI: https://doi.org/10.6084/m9.figshare.21399117.v1.

## Ethics statement

The studies involving human participants were reviewed and approved by Ethics Committee of Beijing Ditan Hospital affiliated to Capital Medical University (#2015-027-01). The patients/participants provided their written informed consent to participate in this study.

## Author contributions

LC designed research. JZ conducted research, recruited and visit subjects. BL performed statistical analysis and wrote paper. YL collected samples and performed data acquisition. TJ conducted research. All authors contributed to the article and approved the submitted version.

## Funding

This work was supported by National Natural Science Foundation of China (Grant No. 32072191), National Key R&D Program of China (Grant No. 2021YFD2100700), Guangxi Science and Technology Project (AD20297088), Daxing District Major Scientific and Technological Achievements Transformation Project (Grant No. 2020006), and Beijing Science and Technology Plan (Grant No. Z201100002620005).

## Conflict of interest

BL, JZ, YL, WQ, and LC were employed by the company Beijing Sanyuan Foods Co. Ltd.

The remaining author declares that the research was conducted in the absence of any commercial or financial relationships that could be construed as a potential conflict of interest.

## Publisher’s note

All claims expressed in this article are solely those of the authors and do not necessarily represent those of their affiliated organizations, or those of the publisher, the editors and the reviewers. Any product that may be evaluated in this article, or claim that may be made by its manufacturer, is not guaranteed or endorsed by the publisher.

## Footnotes

^1^ http://www.yyjsq.cn/
